# Automatic detection of sleep apnea events based on inter-band energy ratio obtained from multi-band EEG signal

**DOI:** 10.1049/htl.2018.5101

**Published:** 2019-06-03

**Authors:** Suvasish Saha, Arnab Bhattacharjee, Shaikh Anowarul Fattah

**Affiliations:** Department of Electrical and Electronic Engineering, Bangladesh University of Engineering and Technology, Dhaka, Bangladesh

**Keywords:** medical signal processing, sleep, medical disorders, electroencephalography, feature extraction, medical signal detection, nearest neighbour methods, signal classification, automatic detection, sleep apnoea events, multiband EEG signal, electroencephalography signal analysis, subject-specific classification, nonapnoea events, sleep disorder, apnoea patient, interband energy ratio features, K-nearest neighbourhood classifier

## Abstract

Sleep apnea is a potentially serious sleep disorder characterised by abnormal pauses in breathing. Electroencephalogram (EEG) signal analysis plays an important role for detecting sleep apnea events. In this research work, a method is proposed on the basis of inter-band energy ratio features obtained from multi-band EEG signals for subject-specific classification of sleep apnea and non-apnea events. The *K*-nearest neighbourhood classifier is used for classification purpose. Unlike conventional methods, instead of classifying apnea patient and healthy person, the objective here is to differentiate apnea and non-apnea events of an apnea patient, which makes the task very challenging. Extensive experimentation is carried out on EEG data of several subjects obtained from a publicly available database. Comprehensive experimental results reveal that the proposed method offers very satisfactory classification performance in terms of sensitivity, specificity and accuracy.

## 1 Introduction

Apnea is a sleep disorder which causes sleep deprivation and lessens sleep quality. It yields severe headaches, hypertension, daytime sleepiness, diminished neurocognitive performance and cardiovascular diseases [1, 2]. It is defined by one or more pauses in breathing or shallow breaths during sleep. Duration of each pause can vary from a few seconds to several seconds. Apnea and hypopnea index (AHI) is defined as the number of occurrences of apnea and hypopnea events per hour of sleep. Generally, AHI of healthy subject should be <5; 5–15 corresponds to mild; 15–30 is moderate; and more than 30 corresponds to severe [3]. Polysomnography (PSG) is a type of sleep study which is used for the diagnosis of sleep apnea. It records the biophysiological changes occurring during sleep. It monitors body functions and several signals such as electroencephalogram (EEG), electro-oculogram (EOG), electrocardiogram (ECG), electromyogram (EMG), oro-nasal airflow, ribcage movements, abdomen movements and oxygen saturation are recorded. Sleep-disordered breathing events, different sleep stages, arousals in sleep etc., are annotated by a sleep technologist. AHI is calculated from the results of PSG study [4].

A sleep technologist is required to monitor and diagnose sleep apnea events, which is a laborious and costly process. Several researches have been pursued to develop automatic apnea detection process utilising different bio-signals including EEG, EOG, ECG and EMG [5]. Among them, the EEG signal has received much attention. The reason behind this is EEG signals reflect the electrical activity of brain, which has a significant relationship with sleep stages and sleep quality, and apnea events acutely disturb sleep quality.

Most of the methods available in the literature deal with the task of classifying apnea patients and healthy subjects [5–8]. In [5], EEG signal is used along with other physiological signals (EOG and EMG) for scoring sleep stages, and to detect apnea frames, ECG signal is used. Here, various time- and frequency-domain feature extraction methods are utilised, namely derivative dynamic time warping, Fourier and wavelet transform and waveform recognition. Instead of using several physiological signals, only EEG signal is also used to detect sleep apnea as it offers less computational cost. The detrended fluctuation analysis of EEG signal is employed in [6] for the purpose of apnea detection. Feature extraction from different frequency bands of EEG signal is a popular technique. In [7], quadratic phase coupling in each frequency band is calculated over bispectral density of EEG, whereas in [8], energy and variance of each frequency band of EEG signal are used to detect apnea frames. Instead of differentiating healthy and apnea subjects, detection of apnea events within an apnea patient is also an important but difficult task. Duration of obstructive sleep apnea event within patient's overnight EEG data is detected in [9] utilising the variation in Hilbert spectrum frequency in particular frequency bands. In [10], the expectancy of identifying sleep-disordered breathing events within an apnea patient is studied analysing the characteristics of EEG frequency bands and EMG signal. In [11], apnea events within an apnea patient are detected using entropy values computed from each frequency band of EEG signal. Sub-frame-based features are extracted and feature variation within a frame is modelled using Rician probability density function (PDF) and model parameters along with some statistical parameters are used in [12] for apnea detection. However, inter-band characteristics have not been utilised in these methods. Moreover, most of the methods [5–8] mentioned above classify between healthy and apnea subjects, while in real-life applications, there is also a great demand for an efficient method to discriminate apnea and non-apnea events of an apnea subject.

The main focus of this research work is to develop an automatic effective sleep apnea event detection method for apnea subject based on inter-band energy ratios of frequency band-limited EEG signals. The study is performed on a standard sleep apnea database. After pre-processing, the energy of band-limited EEG signal is computed for each frequency band of EEG. Instead of using the band energy, inter-band energy ratios are computed and cascaded to construct the proposed feature set. Finally, the *K*-nearest neighbourhood (KNN) classifier is used for apnea and non-apnea classifications.

## 2 Proposed method

**Fig. 1:**
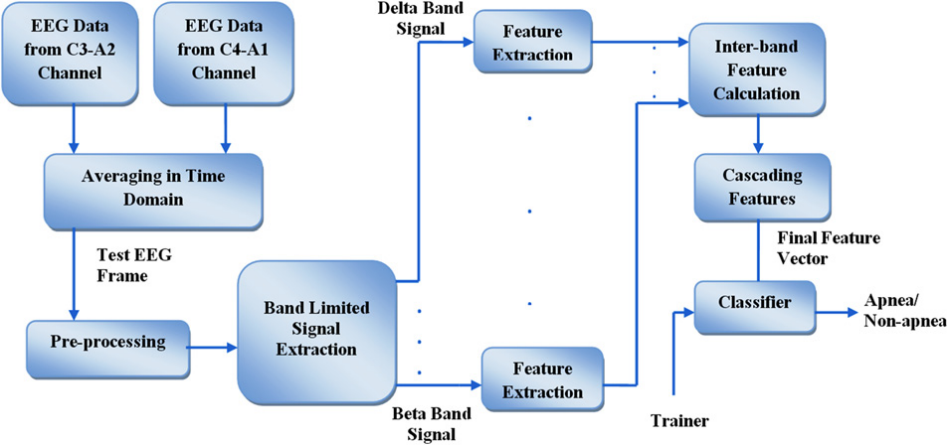
Flowchart of the proposed method

In the proposed method, frame-by-frame apnea detection task is carried out on each subject individually. From given two channel EEG data collected from symmetric locations, in order to reduce the effect of noise, average values of the two channels in time domain are considered for feature extraction in the proposed scheme. Main steps signifying the proposed method are illustrated in Fig. 1. Pre-processed averaged EEG data is divided into band-limited signals and features are extracted from each of them. In lieu of using band features, inter-band features are calculated and cascaded to detect apnea events using the KNN classifier.

### 2.1 Pre-processing

The dc offset of an EEG test frame is eliminated by subtracting the mean value of that frame from each sample value since other frequency components are the significant ones. Frame amplitude normalisation is required to remove undesirable fluctuation in amplitude occurring in different test frames belonging to the same class. For this purpose, after mean value subtraction, normalisation of sample values in a test frame is accomplished with reference to the maximum sample value of that frame.

### 2.2 Band-limited signal extraction

When a person is sleeping, due to pause in breathing, carbon dioxide may build up in the bloodstream. When this reaches a critical level, it is detected by chemoreceptors. These receptors signal an alarm to the brain to wake the sleeping person and breathe in air. As a result, a transition in sleep stages occurs, which in turn, causes fluctuation in activity level of various frequency bands of the EEG signal. Hence, more distinct features can be preserved in frequency band-limited signals for apnea detection compared with that in full-band EEG signal. As a result, for apnea event detection, characteristics of band-limited signals are used instead of analysing full-band EEG signal. EEG signal is partitioned into five frequency bands [10] including delta (*δ*) (0.25–4 Hz), theta (*θ*) (4–8 Hz), alpha (*α*) (8–12 Hz), sigma (*σ*) (12–16 Hz) and beta (*β*) (16–40 Hz). Spectral filtering is done in fast Fourier transform domain to achieve this division.

### 2.3 Proposed inter-band energy ratio feature

For classification of apnea and non-apnea events, a subject-specific classification scheme based on feature is introduced in this proposed method. Inter-band energy ratio of band-limited EEG signal, expected to possess differentiating characteristic for apnea and non-apnea events, is proposed as the feature.

First, energies of *δ*, *θ*, *α*, *σ* and *β* frequency bands are computed. Energy of the *p*th band is defined as

(1) $$E_p = \sum\limits_{n = 1}^N \{{x_p\lsqb n\rsqb } \}^2\comma \; \eqno\lpar 1\rpar $$

where *p* is the band index (i.e. *p* = 1 to 5 corresponds to five frequency bands); *x_p_*[*n*] is the EEG signal of the *p*th band and *N* is the total sample number of a frame.

Next, the inter-band energy ratios are computed. The ratios are: $\delta -\theta $, $\delta -\alpha $, $\delta -\sigma $, $\delta -\beta $, $\theta -\alpha $, $\theta -\sigma $, $\theta -\beta $, $\alpha -\sigma $, $\alpha -\beta $ and $\sigma -\beta $ ratios. The ratio *p–q* is defined as

(2) $$R_{\,pq} = \displaystyle{{E_p} \over {E_q}}\comma \; \eqno\lpar 2\rpar $$

where *p*≠*q*; *p* = 1, 2, 3, 4, 5; *q* = 1, 2, 3, 4, 5; *Ep* and *Eq* correspond to energies of *p*th and *q*th bands of a frame of EEG data, respectively. The calculated features are then cascaded to form the final feature vector.

Energy contents of EEG signal vary in various frequency bands. Lower-frequency bands contain the major percentage of energy during sound sleep (non-apnea event). During apnea, the energy contents in various frequency bands change significantly with respect to non-apnea events. It is found that during apnea events, higher-frequency bands have greater relative energy contribution than that of lower-frequency bands. A hypothesis can be developed based on this observation that energy contents of lower-frequency bands are shifted to higher-frequency bands during apnea events. With a view to investigate this property of energy shift in apnea frames, it is proposed to use all the inter-band energy ratios as features. Ratio feature offers robustness against random energy changes in different frames.

Since energy is mainly shifted from lower- to higher-frequency bands, the energy ratios involving lower- and higher-frequency bands are supposed to be more significant. Hence five ratios, namely $\delta -\theta $, $\delta -\alpha $, $\delta -\sigma $, $\delta -\beta $ and $\theta -\alpha $ are proposed as a reduced feature set. In the proposed method (Prop.), all the ten energy ratios are taken as features and in the proposed method with reduced feature dimension, termed as PRF method, the previously mentioned five significant energy ratios are used as features.

**Fig. 2:**
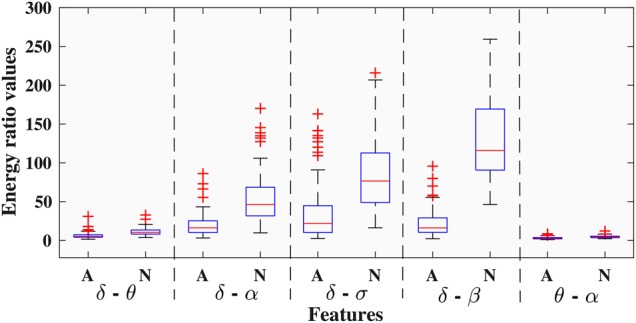
Box plot to show the variation of five energy ratio values for apnea and non-apnea events

To present the quality of energy ratio features, 100 EEG frames for each of the apnea and non-apnea classes obtained from a particular subject are taken into consideration. Fig. 2 represents the box plot of five energy ratio values ($\delta -\theta $, $\delta -\alpha $, $\delta -\sigma $, $\delta -\beta $ and $\theta -\alpha $) of the above-mentioned EEG frames. It is vivid from this figure that majority of the apnea (labelled as ‘A’) and non-apnea (labelled as ‘N’) frames have distinct energy ratio values. The first four ratios have much better distinguishability between ‘A’ and ‘N’ cases. It has been observed that distinguishability tends to decrease for the other ratios.

**Fig. 3:**
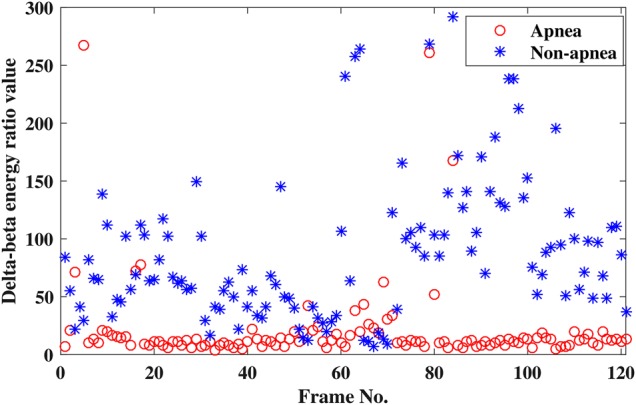
Variation of delta-beta energy ratio values for apnea and non-apnea events

The frame-wise variation of a particular energy ratio value, namely $\delta -\beta $ ratio, is illustrated in Fig. 3 for 121 apnea and non-apnea frames for a particular subject. It is noted from this figure that most of the apnea and non-apnea frames possess distinct $\delta -\beta $ energy ratio values. For some EEG frames, overlaps are also observed in Fig. 3, which are within admissible limit. Depending on the shifting of energy, apnea and non-apnea frames having analogous values for a particular energy ratio are supposed to exhibit distinguishable values for other energy ratios. It is expected that the proposed inter-band energy ratio features can provide satisfactory classification performance because of their good feature quality.

### 2.4 Classification

The KNN classifier is used for classification purpose. Features of the EEG pattern of the test set and *K* neighbouring EEG patterns in the training set are used for computing distance function for the classifier. On the basis of the *K* closer patterns’ class labels in the train set, the test set is classified. Out of several distance functions, in the proposed method, cosine distance is used. The *K* value is changed within a wide range and consistent performance is achieved for all *K* values because of better feature quality. A suitable value of *K* is selected for classification purpose. *M*-fold cross-validation technique is employed for the purpose of performance evaluation.

## 3 Results and discussion

### 3.1 Database and simulation setup

The task of apnea detection is performed on the publicly available Physionet database [13] related to some sleep-disordered breathing subjects diagnosed formerly. In this database, EEG recordings from two channels (C3–A2 and C4–A1) are available. Both channels are considered in this research. The sampling frequency of the EEG data is 128 samples/s. Onset time and duration of apnea events, annotated by a sleep technologist, are mentioned in the database.

Five subjects with large differences in AHI are considered in this research for experimental analysis. The average height, weight and age of the five subjects are 178.6 cm, 99.14 kg and 50.8 year, respectively. Some more information about the subjects and the number of frames considered for each subject are shown in Table 1. For a particular subject, all the apnea frames and equal number of non-apnea frames are taken into consideration for the purpose of evaluating the classification performance. Duration of apnea event is found to vary between 10 and 25 s in most of the cases. To ensure the fact that a frame consists of only apnea or non-apnea condition, a frame duration of 10 s is considered in this experiment.

**Table 1:** Information of the subjects considered in the experiment

Subject number	AHI	Total number of frames
UCDDB003	51	788
UCDDB011	8	88
UCDDB020	15	198
UCDDB024	24	390
UCDDB026	14	242

### 3.2 Feature quality analysis

The quality of the proposed feature is analysed on the basis of geometrical separability index (GSI) which is a standard parameter to understand class separability. The fraction of a set of data points, having the same classification labels as those of their nearest neighbours, is defined as GSI. It is defined as [14]

(3) $$s = \displaystyle{{\sum\nolimits_{i = 1}^N {\lpar f\lpar x_i\rpar + f\lpar {{x}^{\prime}}_i\rpar + 1\rpar \bmod \, 2} } \over N}\comma \; \eqno\lpar 3\rpar $$

where *N* is the number of points and ${x}^{\prime}$ denotes the nearest neighbour of *x*. It is clear that *s* will approximate to 1 when a set of data points having opposite classification labels reside in clusters which are well-separated. The index will decrease when the data points having opposite classes start to overlap defining poorly-separated clusters. Hence, higher GSI value defines better quality feature.

GSI values of the proposed method (Prop.) and PRF method are represented in Table 2. It is clearly observed from this table that both the proposed method and PRF method have higher GSI values. Thus, the proposed feature performs better in classifying apnea and non-apnea events. The area under the receiver operating characteristic (ROC) curve (AUC) is a measure of how well a parameter can distinguish between two diagnostic groups (diseased/normal). Higher AUC value indicates better classifier performance. The AUC values computed for the methods with the KNN classifier are represented in Table 2. It is observed that both the methods have higher AUC values.

**Table 2:** GSI and AUC values of different methods

Subject number	GSI value		AUC value	
	Prop.	PRF	Prop.	PRF
UCDDB003	0.85	0.85	0.95	0.94
UCDDB011	0.90	0.92	0.95	0.94
UCDDB020	0.95	0.94	0.99	0.99
UCDDB024	0.91	0.92	0.97	0.96
UCDDB026	0.82	0.83	0.92	0.91
mean	0.89	0.89	0.96	0.95
standard deviation	0.05	0.05	0.02	0.03
interquartile range	0.08	0.08	0.03	0.04

### 3.3 Classification result

The performance of the proposed method is evaluated using standard measures such as accuracy, sensitivity and specificity, which are defined as

(4) $${\rm accuracy} = \displaystyle{{{\rm TP} + {\rm TN}} \over {{\rm TP} + {\rm TN} + {\rm FP} + {\rm FN}}} \times 100\eqno\lpar 4\rpar $$

(5) $${\rm sensitivity} = \displaystyle{{{\rm TP}} \over {{\rm TP} + {\rm FN}}} \times 100\eqno\lpar 5\rpar $$

(6) $${\rm specificity} = \displaystyle{{{\rm TN}} \over {{\rm TN} + {\rm FP}}} \times 100\comma \; \eqno\lpar 6\rpar $$

where TP is the true positive (apnea detected as apnea); FP is the false positive (non-apnea detected as apnea); TN is the true negative (non-apnea detected as non-apnea); and FN is the false negative (apnea detected as non-apnea).

Classification results obtained by averaging the individual sensitivity, specificity and accuracy of the five mentioned subjects for different channel data for the proposed method (Prop.) and PRF method are reported in Table 3 for leave-one-out cross-validation scheme. It is observed from this table that, instead of considering single channel, if the average (avg.) data of the two channels (C3–A2, C4–A1) are considered for feature extraction, better classification performance is achieved. In method PRF, only five features are used compared with the ten features of the proposed method. Hence, the computational complexity is reduced a bit which can be helpful in real-time application. It is observed from Table 3 that the performance of the PRF method is slightly better in one case and descends slightly for the other two cases with respect to the proposed method.

**Table 3:** Comparison of apnea detection results for different channel data

Channel number	Sensitivity, %		Specificity, %		Accuracy, %	
	Prop.	PRF	Prop.	PRF	Prop.	PRF
C3–A2	84.96	84.63	90.88	91.61	87.92	88.12
C4–A1	89.37	88.46	92.20	90.69	90.78	89.57
average data	90.39	89.70	94.04	93.22	92.21	91.46

The average classification results for the proposed method and PRF method using different classifiers are represented in Table 4 for leave-one-out cross-validation scheme. It is observed from this table that the KNN classifier gives better performance.

**Table 4:** Comparison of classification results for different classifiers

Classifier	Prop.			PRF		
	Sens.	Spec.	Acc.	Sens.	Spec.	Acc.
KNN	90.39	94.04	92.21	89.70	93.22	91.46
support vector machine	91.10	84.81	87.95	90.76	80.99	85.87
linear discriminant analysis	90.74	89.42	90.08	93.39	72.73	83.06
Naïve Bayes	90.74	89.42	90.08	91.28	82.31	86.80

The average classification results for different distance functions of KNN classifier are reported in Table 5 for leave-one-out cross-validation scheme. It is observed from this table that the classification result does not depend much on distance function but the cosine distance gives comparatively better result.

**Table 5:** Comparison of classification results for different distance types

Distance	Average accuracies, %	
	Prop.	PRF
Euclidean	89.11	89.75
Cityblock	89.45	89.54
Cosine	92.21	91.46
Correlation	92.21	88.06

The performance comparison of the proposed method with the existing methods reported in [8, 11, 12] is provided in Table 6 for leave-one-out cross-validation scheme. In [8], energy and variance of each frequency band of EEG data are used as features to distinguish between controls and patients. The method reported in [11] utilises entropy values obtained from five frequency bands of EEG data to detect apnea events. The method reported in [12] utilises sub-frame-based feature extraction for apnea detection, which involves huge computational burden in comparison with the proposed method, where no sub-framing is performed. Moreover, the number of features used in [12] is also much higher in comparison with that used in the proposed method (four times higher with respect to the proposed method). It is to be noted that for fair comparison, frame duration of 10 s and KNN classifier are used in all cases. In [12], for the database [13], 90% overlap between two successive frames is considered, and sub-frame duration is chosen as two-thirds of the original frame duration (15 s). The same principle is applied on the 10 s frames for the method in [12] for comparison purpose. It is observed from Table 6 that the performance of the proposed method is better than that of the existing methods with respect to all the standard performance criteria. The confusion matrix of the classification for the five subjects considered in this Letter for the proposed method for leave-one-out cross-validation scheme is given in Table 7.

**Table 6:** Performance comparison among various methods of apnea event detection

Subject number	Sensitivity, %				Specificity, %				Accuracy, %			
	[8]	[11]	[12]	Prop.	[8]	[11]	[12]	Prop.	[8]	[11]	[12]	Prop.
UCDDB003	87.31	79.19	81.22	87.56	88.58	81.73	87.31	88.58	87.94	80.46	84.26	88.07
UCDDB011	86.36	90.91	86.36	88.64	72.73	88.64	84.09	97.73	79.55	89.77	85.23	93.18
UCDDB020	82.83	84.85	86.87	96.97	94.95	90.91	95.96	97.98	88.89	87.88	91.41	97.47
UCDDB024	85.13	92.31	91.28	92.82	90.77	91.79	93.85	93.33	87.95	92.05	92.56	93.08
UCDDB026	85.95	80.99	85.95	85.95	90.08	86.78	90.08	92.56	88.02	83.88	88.02	89.26
mean	85.52	85.65	86.34	90.39	87.42	87.97	90.26	94.04	86.47	86.81	88.30	92.21
standard deviation	1.70	5.83	3.57	4.47	8.55	4.00	4.80	3.93	3.89	4.65	3.66	3.72
interquartile range	2.05	10.72	3.20	6.70	7.20	5.62	7.87	6.22	2.39	7.31	6.72	5.30

**Table 7:** Confusion matrix of classification

Subject number	UCDDB003		UCDDB011		UCDDB020		UCDDB024		UCDDB026	
Label	Apnea	Non-apnea	Apnea	Non-apnea	Apnea	Non-apnea	Apnea	Non-apnea	Apnea	Non-apnea
apnea	345	49	39	5	96	3	181	14	104	17
non-apnea	45	349	1	43	2	97	13	182	9	112

Comparison of mean classification results for leave-one-out, ten-fold, five-fold and two-fold cross-validation methods are reported in Table 8 to represent the regularity of the classification results in case of random variation in the training and test set data. It is observed that the proposed method gives better performance than the existing methods in all cases.

**Table 8:** Comparison of classification results for different cross-validation methods

Cross-validation	Sensitivity, %				Specificity, %				Accuracy, %			
	[8]	[11]	[12]	Prop.	[8]	[11]	[12]	Prop.	[8]	[11]	[12]	Prop.
leave-one-out	85.52	85.65	86.34	90.39	87.42	87.97	90.26	94.04	86.47	86.81	88.30	92.21
10-fold	85.74	85.12	86.22	90.84	88.09	87.43	91.25	93.69	86.59	86.36	88.90	92.02
5-fold	86.52	87.12	86.67	89.90	87.40	87.95	91.27	93.75	86.91	87.71	88.84	91.59
2-fold	84.67	79.31	86.99	89.46	85.90	85.14	88.67	89.40	85.23	82.12	87.44	89.37

## 4 Conclusion

In this research work, an automatic efficient sleep apnea event detection scheme is proposed based on inter-band energy ratios of multi-band EEG signal. Features are extracted from time-averaged data of two EEG channels, which helps in reducing the effect of noise. Band energy of EEG data may change randomly but inter-band energy ratio feature proposed in this Letter offers robustness with better feature quality. The proposed method offers superior subject-specific classification performance compared with that achieved by some recent methods in terms of sensitivity, specificity and accuracy for sleep apnea subjects with wide variation in AHI index.
